# Complete mitochondrial genome of scorpionfish *Scorpaena neglecta* (Actinopterygii)

**DOI:** 10.1080/23802359.2022.2100292

**Published:** 2022-07-28

**Authors:** Maheshkumar Prakash Patil, Jong-Oh Kim, Yong Bae Seo, Jiyoung Shin, Ji-Young Yang, Gun-Do Kim

**Affiliations:** a Industry-University Cooperation Foundation, Pukyong National University, Busan, Republic of Korea; b Department of Microbiology, Pukyong National University, Busan, Republic of Korea; c School of Marine and Fisheries Life Science, Pukyong National University, Busan, Republic of Korea; d Research Institute for Basic Science, Pukyong National University, Busan, Republic of Korea; e Institute of Food Science, Pukyong National University, Busan, Republic of Korea; f Department of Food Science and Technology, Pukyong National University, Busan, Republic of Korea

**Keywords:** *Scorpaena neglecta*, mitochondrial genome, phylogenetic analysis, Scorpaenidae, Scorpaeniformes

## Abstract

*Scorpaena neglecta* (Temminck and Schlegel, 1843) is a marine fish, in the family Scorpaenidae, order Scorpaeniformes, class Actinopterygii of the phylum Chordata. The first species of *Scorpaena* with a complete mitochondrial genome is described in the present study. The circular mitochondrial genome of *S. neglecta* has 17,202 bp with 54.75% A + T content and encodes 37 genes, including 13 protein-coding genes (PCGs), 22 transfer RNA (tRNA), and two ribosomal RNA (rRNA). The phylogenetic tree indicates *S. neglecta* clustered into one branch and is closely related to other Scorpaenidae species. The mitochondrial genome structure and gene content of *S. neglecta* will support the study of evolution and phylogenetic relationships among Scorpaenidae species.

*Scorpaena neglecta* belongs to the class Actinopterygii and the family Scorpaenidae (also known as scorpionfish) and is mainly distributed in South Korea and Japan (Moon et al. 2016). Scorpionfish are venomous marine fish found in the Indo-Pacific Ocean, including Indonesia, Japan, South Korea, Philippines, Taiwan, and Australia. Different species of these fish have similar morphologic traits, making identification difficult (Wibowo and Motomura 2019). However, there is little knowledge of its mitochondrial genetic features. As a result, we focused our research on the mitochondrial genome of *S. neglecta* and their evolutionary connection within the Scorpaenidae family.

A specimen of *S. neglecta* was collected from the coast of Jeju, South Korea (33°15′59.1840″N 126°42′18.4638″E), and a voucher specimen (no.: MFDS-FBO10) was deposited at the Food Engineering Department, Pukyong National University, Busan, South Korea (Ji-Young Yang, jyyang@pknu.ac.kr). From muscle tissue, total DNA was extracted according to the manufacturer's instructions using the DNeasy Blood and Tissue Kit (Qiagen, Hilden, Germany). The DNA library was made using the TrueSeq Nano DNA Kit and sequenced with paired-end reads (150 bp) on the Illumina platform (Illumina HiSeq 2500, San Diego, CA). Next, the Platanus-allee v2.2.2 assembly tool (Kajitani et al. 2019) was used for *De novo* assembly and the MitoFish web-server (http://mitofish.aori.u-tokyo.ac.jp/) was used for complete mitochondrial genome sequence annotation (Iwasaki et al. 2013). A phylogenetic tree was built in MEGA11 using the maximum-likelihood approach (Tamura–Nei model, 1000 bootstraps) (Tamura et al. 2021) using the complete mitochondrial genome sequences of Scorpaenidae members retrieved from NCBI (https://www.ncbi.nlm.nih.gov/).

The mitochondrial genome sequence of *S. neglecta* has been submitted to GenBank under the accession number ON109388.1. The close-circular mitochondrial genome of *S. neglecta* was 17,202 bp long, containing a total of 37 genes, including 22 transfer RNA (tRNA) genes, two ribosomal RNA (rRNA) genes, and 13 protein-coding genes (PCGs). The individual base composition covers A 4878 bp (28.35%), T 4542 bp (26.40%), G 3002 bp (17.45%), and C 4780 bp (27.78%). Out of 37 genes, nine genes (ND6 and eight tRNA (Pro, Glu, Ser, Tyr, Cys, Asn, Ala, and Gln)) were present on L-strand. The 16S and 12S rRNA genes are situated between the tRNA-Leu and tRNA-Phe genes, separated by the tRNA-Val gene, like in other vertebrates. In the present study, we found that the genes including Cyt B, ATPase 6, COIII, COII, ND3, and ND2 have an incomplete stop codon and atypical codon use in tRNA-Leu (UAG, UAA) and tRNA-Ser (GCU, UGA).

The maximum-likelihood phylogenetic tree based on mitochondrial genome sequences of *S. neglecta* and other nine species from the Scorpaenidae family with one outgroup species (*Eopsetta jordani*, OK545541.1) was constructed under the Tamura–Nei model with 1000 bootstraps (Figure 1). In this study, *S. neglecta* (ON109388.1) is placed on a sister branch with *Scorpaenopsis cirrosa* (NC027735.1) and *Scorpaenopsis ramaroi* NMMBP:1280 (LC493915.1) from the same family and reveals a close relationship with other species in a phylogenetic tree. This is the first report on the Scorpaena species, and the complete mitochondrial genome data will be useful in future research on Scorpaenidae species evolution and phylogenetic connections.

**Figure 1.**
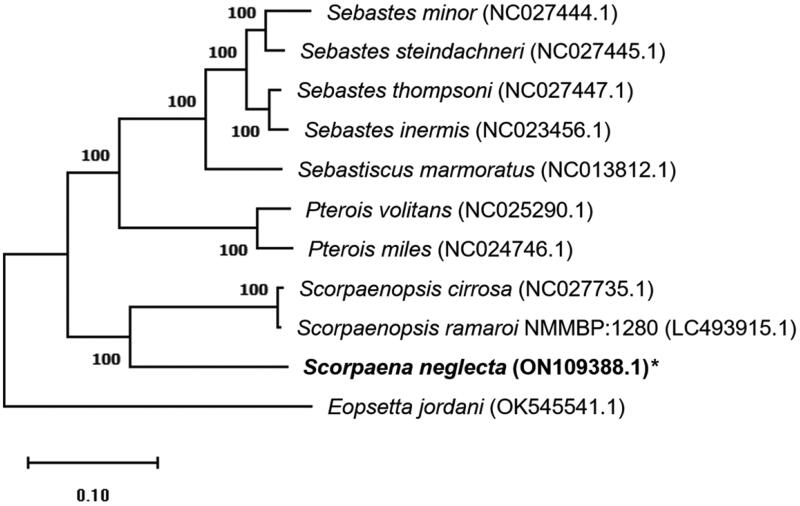
The phylogenetic tree indicates the relationship between *S. neglecta* (ON109388.1) and nine Scorpaenidae species, with *Eopsetta jordani* (OK545541.1) as the outgroup, based on the mitochondrial genome sequence retrieved from the NCBI database. The numbers (%) in each node represent the bootstrap possibilities. The mitochondrial genome determined in this work is indicated by an asterisk after the species name.

## Data Availability

The genome sequence data that support the findings of this study are openly available in GenBank of NCBI at https://www.ncbi.nlm.nih.gov/ under accession no. ON109388. The associated BioProject, BioSample, and SRA numbers are PRJNA826296, SAMN27554322, and SRR18740261, respectively.
